# Unraveling the Impact of *Aspergillus sojae*—A Food-Grade Fungus—On Phytoalexins, Phenolic Acids, and the Antioxidant and Antidiabetic Activity of Different Legumes

**DOI:** 10.3390/foods13223533

**Published:** 2024-11-05

**Authors:** Shalika Rana, William Broussard, Steven Elliott, Matthew E. Burow, Stephen M. Boue

**Affiliations:** 1 Southern Regional Research Center, Agricultural Research Service, U. S. Department of Agriculture, New Orleans, LA 70124, USA; 2 Section of Hematology & Medical Oncology, Tulane Departments of Medicine, New Orleans, LA 70112, USA

**Keywords:** phytoalexins, *Aspergillus sojae*, UPLC, α-glucosidase, lipase, UPLC-ESI-QTOF-MS/MS

## Abstract

Legumes are a rich source of polyphenolic compounds known for their ability to promote health. Under stress conditions, legumes have been shown to produce higher levels of secondary metabolites, as a defensive mechanism. Hence, the present study aimed to induce legume seeds (e.g., soybean, chickpea, green pea, and red kidney bean) by inoculating them with *Aspergillus sojae* (*A. sojae*) and to evaluate the extracts for phytoalexins, phenolics, and antioxidant, antiobesity, and antidiabetic potentials. The UPLC-DAD findings of *A. sojae*-induced legumes showed medicarpin and maackiain in chickpea, pisatin in green pea, glyceollin I-III in soybean, and kievitone and phaseollin in red kidney bean. All induced legumes exhibited a higher total polyphenol content compared to the non-induced ones. Among induced legumes, soybean exhibited a higher (4.85 mg GAE/g) polyphenol content. The UPLC-ESI-QTOF-MS/MS findings established that legumes contained substantial levels of protocatechuic acid, vanillic acid, ferulic acid, chlorogenic acid, coumaric acid, 4-hydroxybenzoic acid, and caffeic acid. The results of antioxidant assays revealed a significantly higher level of activity in induced red kidney bean and soybean, whereas the level of activity in non-induced legumes was markedly lower. Moreover, induced red kidney bean effectively inhibited α-glucosidase (87.2%) and α-amylase (63.90%) at 5 mg/mL. Additionally, the maximum lipase inhibitory effects were displayed by induced soybean (72.54%) at 20 mg/mL.

## 1. Introduction

*Aspergillus sojae* is a fungal strain that is commonly used in the production of many foods, particularly in the preparation of fermented soybean products [1,2]. This fungus is considered as a safe food ingredient and is commonly used for the preparation of soy sauce in Japan [1]. Legumes, including soybean, are included in many diets worldwide. They are a good source of protein and contain many polyphenolic compounds that promote health. Polyphenols consist of several classes of compounds that include phenolic acids, flavonoids, and tannins [3,4]. The most common polyphenols identified in legumes are phenolic acids that are separated into hydroxycinnamic acids and hydroxybenzoic acids. Flavonoids are also among the common polyphenols in legumes, and they affect the color and flavor of common beans [4]. In response to stress, compounds called phytoalexins are produced by plants, which are a part of the plant’s defense system [5]. There are several reports showing that phytoalexins are produced in legumes under conditions of stress [6,7]. Phytoalexins are produced in plants after infection by fungi; however, other biotic elicitors have also been shown to induce plant phytoalexins [8]. Research has shown that elicitor treatments can increase the polyphenol content in legumes [9]. There are generally two methods used for producing phytoalexins in legumes, i.e., abiotic and biotic elicitors. One study has shown that peanuts produce resveratrol when challenged with *Aspergillus caelatus* [10]. Research in our laboratory has shown that soybean seeds produce the isoflavone phytoalexins called glyceollins after elicitor treatment [11]. It has been proven that glyceollin isomers I-III were absent in non-inoculated soybean cotyledon tissue, but after A. sojae inoculation, the content of glyceollin isomers I-III in cotyledon tissue was 1117 µg/g [11].

Polyphenols distinctively contribute to the health-promoting potential of legumes, offering an array of health benefits. Several beneficial properties of legume polyphenols have been observed, including improving cardiovascular health, suppressing the onset of diabetes, exhibiting anticancer activity, and improving glucose homeostasis [12,13]. Additionally, polyphenols in legumes can provide antioxidants that protect the body’s cells from harmful reactive oxygen species and different free radicals. Several studies have examined the antioxidant activities of different legumes [14,15]. Zhao et al., studied the total antioxidant activity of different legumes, and the level of activity of the legumes was in the following order: chickpea > small red kidney bean > black kidney bean > cowpea > navy bean > baby lima bean [15]. Moreover, legumes have been shown to possess therapeutic antiobesity and antidiabetic effects by inhibiting the enzymes involved in carbohydrate and fat metabolism. Researchers detailed the ability of legume extracts from Vigna species to inhibit α-glucosidase and lipase activities [16].

Diabetes and obesity are the primary sources of morbidity in the United States with major economic impacts [17,18]. Several studies have indicated that obesity is linked to a higher risk of diabetes [19]. A high-fat diet and fat accumulation are major problems in many countries, and the development of safe and effective treatments is often difficult and expensive. Diabetes is defined by increased blood glucose (hyperglycemia) which causes higher levels of harmful reactive oxygen species and free radicals. These harmful components can lead to health complications like cardiovascular disease and nerve damage [20,21]. One method for managing diabetes is to target the digestive enzymes α-amylase and α-glucosidase by using plant extracts and components to inhibit the breakdown of starch into glucose [22]. Long-chain carbohydrates are broken down by α-amylase that acts at random sites along the starch chain, leading to the production of maltotriose and maltose [22]. α-glucosidase is located in the small intestinal epithelium, where oligosaccharides and disaccharides are converted into glucose. Blocking both digestive enzymes, α-amylase and α-glucosidase, causes a slower rate in carbohydrate digestion, thus suppressing postprandial hyperglycemia [22,23]. Currently, there are several glucosidase inhibitors (including acarbose, miglitol, and voglibose) that are prescribed to individuals with diabetes, but these commercial inhibitors can have several harmful side effects [24,25]. Recent research has also targeted treatments for obesity. One strategy to treat obesity is blocking the enzyme pancreatic lipase that decreases intestinal fat absorption. The drug orlistat, a derivative of lipstatin produced by *Steptomyces toxytricini*, inhibits dietary fat absorption by 30% and reduces body weight in patients. Unfortunately, orlistat has several undesirable side effects [26], and these problems have created demand for alternative treatments. Many plant extracts, including legumes, are rich in polyphenols and have been shown to inhibit lipase activity [16,27,28,29,30,31].

Although the polyphenols in legumes have been shown to have the potential to provide various health benefits, more information is needed on the ability of stress-induced legume extracts and phytoalexins to inhibit digestive enzymes. Few studies have been carried out on the induction of legume polyphenols and antioxidant activities using *A. sojae*. Our laboratory research has recently focused on the ability of polyphenols in legume extracts to inhibit digestive enzymes. Here, we report the ability of four legume extracts with and without *A. sojae* treatment to inhibit several digestive enzymes: α-amylase, α-glucosidase, and pancreatic lipase. All legumes were characterized for total polyphenol and flavonoid content. Additionally, the phenolic acids were quantitated, and the antioxidant activities of each legume extract were examined using different antioxidant assays.

## 2. Materials and Methods

### 2.1. Chemicals and Reagents

Folin–Ciocalteu reagent, AAPH (2,2-axobis-2-methyl-propanimidamide dihydrochloride), 2,2′-azinobis-(3-ethylbenzothiazoline-6 sulfonic acid) (ABTS), 2,2-diphenyl-1-picrylhydrazyl (DPPH), 6-hydroxy-2,5,7,8-tetramethylchroman-2-carboxylic acid (Trolox), porcine pancreatic lipase (type II), α-glucosidase (type I), α-amylase, p-nitrophenyl-α-*D*-glucopyranoside (PNGP), CNPG3 (2-chloro-4-nitrophenyl-α-maltotrioside), 4-methylumbelliferyl oleate (4-MUO), maackiain, medicarpin, quercetin, gallic acid, protocatechuic acid, vanillic acid, ferulic acid, chlorogenic acid, coumaric acid, 4-hydroxybenzoic acid, caffeic acid, and ethanol were obtained from Sigma-Aldrich (St. Louis, MO, USA). Glyceollin I, kievitone, and phaseollin were isolated at the SRRC (New Orleans, LA, USA, 70124) [11,32]. The pisatin standard was purchased from Biosynth (Berkshire, UK).

### 2.2. Induction of Legume Seeds by Food-Grade Fungus A. sojae

#### Fungal Cultures and Fungal Inoculations of Legume Seeds

*Aspergillus sojae* (SRRC 1125) cultures were grown on potato dextrose agar at 25 °C using a method developed in our laboratory [33]. Conidia were collected and suspended in sterile distilled water (15 mL) at a concentration of 1.0–3.0 × 10⁷ conidia/mL. Soybean, chickpea, green pea, and red kidney bean seeds were surface-sterilized using 70% ethanol for 3 min, followed by three water rinses. The seeds were then placed in water for 8 h, then cut using a sterile razor blade and placed in prepared Petri dishes. Each Petri dish (100 × 15 mm) was lined with autoclaved Whatman filter paper, and 500 µL water was added. An *A. sojae* spore suspension (10 µL) was applied to each cut legume seed. Control seeds were uncut and used without soaking in water. All Petri dishes were incubated at 25 °C for 3 days in darkness.

### 2.3. Extraction of A. sojae-Induced and Non-Induced Legumes

Legume seeds viz. soybean (SB), chickpea (CP), green pea (GP), and red kidney beans (RKBs) induced by *A. sojae* and those non-induced were milled to obtain fine powder using a Tekmar Mill. Milled samples were passed through a 30 µm sieve to obtain constant size. For the extraction of phenolics, 0.1 g of each legume sample was extracted for 1 h with 1 mL of HPLC-grade methanol by ultrasonication at room temperature. Subsequently, tubes were centrifuged at 12,000× *g* for 15 min to obtain clear supernatants of each extract. Each extracted sample was filtered through a 0.45 μm PVDF membrane. Extracted samples were used to determine phytoalexin, phenolic acids, total phenolics, antioxidant activity, and enzyme inhibitory assays. *A. sojae*-induced green pea, chickpea, soybean, and red kidney bean extracts were labeled as GP-AS, CP-AS, SB-AS, and RKB-AS, respectively, and non-induced (controls) green pea, chickpea, soybean, and red kidney bean extracts were labeled as GP-CON, CP-CON, SB-CON, and RKB-CON, respectively.

### 2.4. UPLC-DAD Quantification of Phytoalexins

To determine the content of phytoalexins in legume samples, a Waters UPLC-DAD system was used. The separation of all the phytoalexins was achieved using a Waters Acquity BEH C-18 column (100 mm × 2.1 mm, 1.7 mm). A 5 µL injection volume was used, and the flow rate of the mobile phase was 0.300 mL/min. The mobile phase used for the separation was (A) acetonitrile and (B) 0.1% formic acid in water. Linear calibration curves (R^2^ > 0.999) for each phytoalexin were prepared in a range of 4–200 µg/mL. The wavelengths used for quantitation were as follows: 285 nm for glyceollins I, II, and III in soybean, phaseollin in red kidney bean, and medicarpin in chickpea; 292 nm for kievitone in red kidney bean; and 310 nm for maackiain in chickpea and pisatin in green pea. All the experiments were conducted three times, and the data were reported as the mean ± SD. Phytoalexin concentration was reported as the µg/g of each legume on a dry weight basis.

### 2.5. Total Phenolic Content and Total Flavonoid Content

The total phenolic content in the *A. sojae*-induced and non-induced legume samples for red kidney bean, chickpea, soybean, and green pea was determined using the Folin–Ciocalteu reagent method [34]. Briefly, 20 µL aliquot of the sample was added to 100 µL Folin–Ciocalteu reagent (1:10) in a 96-well microplate. Plates were shaken and incubated at room temperature for 10 min. Subsequently, 100 µL of 7.5% sodium carbonate was added into the wells, and plates were incubated at room temperature for 10 min. The absorbance was taken at 750 nm in a microplate reader (Biotek Synergy H1). Gallic acid was employed for the generation of the standard curve. The results of the assay were expressed as mg gallic acid equivalents (GAE)/g of sample.

The total flavonoid content of different legume extracts was quantified according to the method outlined by Mahboubi et al. [35] with some modifications. Using a test tube, 1.9 mL of methanol and 100 µL of the sample was added followed by the addition of 100 µL of 10% aluminum chloride and 100 µL of 1 M potassium acetate. The solution volume was set to 5 mL through the addition of 2.8 mL of methanol, and the solution was incubated at room temperature for 30 min and briefly vortexed. The absorbance of the plate was read at 415 nm using a UV-1900i Shimadzu spectrophotometer. The total flavonoid content of the legumes samples was evaluated using quercetin as the standard. The findings of the assay were displayed as mg quercetin equivalents (QE)/g sample.

### 2.6. UPLC-ESI-QTOF-MS/MS Analysis of Phenolic Acids in Legume Extracts

Protocatechuic acid, vanillic acid, ferulic acid, chlorogenic acid, coumaric acid, 4-hydroxybenzoic acid, and caffeic acid were analyzed using UPLC-ESI-QTOF-MS/MS. Phenolic acid analysis were performed utilizing a Waters Acquity UPLC instrument and a Xevo G2-XS QTOF mass spectrometer (Waters Corp., Milford, MA, USA) equipped with an electrospray (ESI) source used in negative ionization mode. A Waters Acquity BEH C-18 column (150 mm × 2.1 mm, 1.7 mm) was used, and the temperature of the column chamber was set to 50 °C. The mobile phase used was composed of (A) 0.1% formic acid in water and (B) 0.1% formic acid in acetonitrile with a flow rate of 0.3 mL/min. The gradient elution program was as follows: 0–0.3 min, 3% B; 0.3 to 9 min linear gradient to 95% B; 9–12 min 95% B; 12–13 min linear gradient to 3% B; and 13–15 min 3% B. The injection volume was 2 µL. The mass spectrometer settings used are as follows: sampling cone 15 V, temperature 120 °C, capillary voltage 0.8 kV, extraction cone 4.0 V, source collision energy (ramp 30–35 eV), and desolvation gas flow rate 600 L/h. Multiple Reaction Monitoring (MRM) mode was utilized with a specific target to product ion transition at a specific time interval related to the retention time of the analytes. Each target ion and retention time were selected from standards, and quantitation was achieved using the most abundant product ions.

### 2.7. Antioxidant Capacity of A. sojae-Induced and Non-Induced Legumes

#### 2.7.1. Oxygen Radical Absorbance Capacity (ORAC) Assay

The oxygen radical absorbance capacity assay was conducted in a 96-well plate [36]. The ORAC assay follows the decline in fluorescein fluorescence due to the generation of peroxyl radicals from the breakdown of AAPH (2,2-axobis-2-methyl-propanimidamide dihydrochloride). A microplate reader (Biotek Synergy H1) was used for readings with an excitation wavelength at 485 nm and emission wavelength at 528 nm, respectively. Trolox was used as a positive control for the assay. The results of the ORAC assay were displayed as µmols Trolox equivalents (TE)/g of sample.

#### 2.7.2. DPPH Radical Scavenging Activity

The DPPH free radical scavenging activity of legume samples was measured by the method described by Ben Mansour et al. [37]. To reveal the free radical scavenging effects of induced and non-induced legumes, the DPPH method was used. In a 96-well plate, a 50 μL aliquot of the respective sample was combined with 150 μL DPPH solution of 200 µM solution. Next, the plates were incubated for 30 min. The microplate reader absorbance was set at 520. The percent inhibition was calculated as

$$\%\;\mathrm{Inhibition}=\frac{Control-Sample}{Control}\times 100$$

A standard curve was generated using Trolox. The results were calculated as mg Trolox equivalents (TE)/g of sample. All the analyses were conducted in triplicate.

#### 2.7.3. ABTS Radical Cation-Based Assay

The ABTS assay was conducted using the method published by Re et al. with some modifications [38]. ABTS (7 mM) and potassium persulfate (2.45 mM) were mixed to prepare the ABTS·+ solution and placed in darkness for 16 h. For the analysis, a 10 μL aliquot of the respective sample was added to each well of a 96-well plate, and then 190 μL of ABTS solution was added to each well. Next, the plate was incubated for 6 min, and the absorbance was measured at 734 nm. All the analyses were conducted in triplicate. The results of the scavenging assay were established as mg Trolox equivalents (TE)/g of sample.

### 2.8. Antidiabetic Assays

#### 2.8.1. α-Glucosidase Enzymatic Inhibition

The α-glucosidase enzymatic inhibition assay was conducted using the method published by Apostolidis et al. [39]. A control, or sample was added to a 96-well plate containing 50 μL of buffer to 100 μL of a 1 U/mL α-glucosidase solution. Both enzyme and substrate solutions were prepared in 0.1 M sodium phosphate buffer, pH 6.9, and then the reaction mixture was incubated for 10 min. Subsequently, 5 mM p-nitrophenyl-α-D-glucopyranoside (50 μL) was placed into each well followed by incubation at 25 °C for 5 min. The absorbance was measured at 405 nm using a microplate reader (Biotek Synergy H1) before and after incubation. The results were calculated accordingly:

$$\%\;\mathrm{Enzyme}\;\mathrm{inhibition}=\frac{Absorbance\;of\;control-Absorbance\;of\;sample}{Absorbance\;of\;control}\times 100$$

#### 2.8.2. α-Amylase Inhibition

The inhibition of α-amylase was measured using the method by Zulfiqar et al. [40]. First, 50 µL of each legume extract was incubated for 10 min at 37 °C with porcine pancreatic α-amylase (100 µL, 1 U/mL) within a 20 mM phosphate buffer at pH 6.9 for in a 96-well plate. Subsequently, a 2 mM CNPG3 (2-chloro-4-nitrophenyl-α-maltotrioside) substrate (50 µL) prepared in phosphate buffer was added. The absorbance was monitored at 405 nm every minute over a 10 min interval using a Biotek Synergy H1 microplate reader. The level of enzyme inhibition (%) was determined based on the difference in each sample’s absorbance relative to the non-inhibited control absorbance.

### 2.9. Pancreatic Lipase Inhibition

Pancreatic lipase inhibition was evaluated using the fluorometric method by Podsedek et al. and Luo et al. [41,42]. All legume samples were dissolved in 13 mM Tris-HCl buffer with 75 mM sodium chloride and 1.3 mM calcium chloride (pH 8.0). Samples (25 µL) were combined with the enzyme solution (25 µL, prepared in the same buffer) and incubated at 37 °C for 5 min. After incubation, the 4-methylumbelliferyl solution (50 µL prepared in buffer) was added. After incubation for 20 min at 37 °C, the fluorescence value was measured using an excitation wavelength of 355 nm and an emission wavelength of 460 nm. Both sample blanks and control blanks (devoid of substrate) were prepared in the same manner. (%) Inhibition was determined with the following equation:

$$\mathrm{Inhibition}\;\left(\%\right)=\frac{\left(F_{control-}F_{control\;blank}\right)-\left(F_{sample-}F_{sample\;blank}\right)}{\left(F_{control-}F_{control\;blank}\right)}\times 100$$

where F_control_ is the fluorescence of the control with substrate, F_control blank_ is the fluorescence of the control devoid of substrate, F_sample_ is the fluorescence of the sample with substrate, and F_sample blank_ is the fluorescence of the sample devoid of substrate.

### 2.10. Statistical Analysis

The results of all the experiments are displayed as the mean ± SD. Statistical differences were analyzed using a one-way ANOVA and two-way ANOVA using Tukey’s multiple comparisons. A *p*-value below 0.05 was considered statistically significant. All statistical assessments were conducted via GraphPad Prism 10.2.3 software.

## 3. Results and Discussion

### 3.1. Characterization of Phytoalexins in A. sojae-Induced Legumes

**Figure 1:**
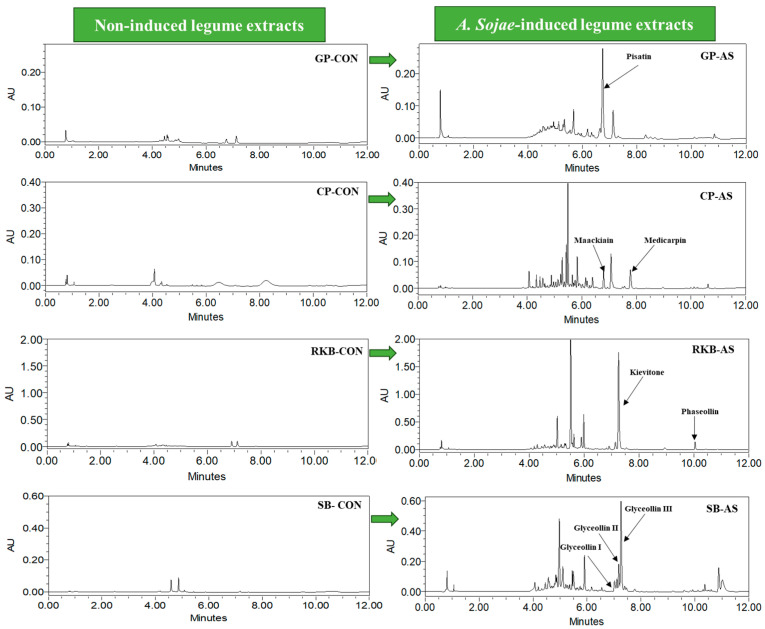
UPLC-DAD chromatogram of phytoalexins in *A. sojae*-induced and non-induced legume extracts. GP-CON, green pea non-induced; GP-AS, green pea induced; CP-CON, chickpea non-induced; CP-AS, chickpea induced; SB-CON, soybean non-induced; SB-AS, soybean induced; RKB-CON, red kidney bean non-induced; RKB-AS, red kidney bean induced. PDA wavelengths used: GP samples at 310 nm; SB, RKB, and CP at 285 nm.

Legumes are a rich source of polyphenols that have been shown to be beneficial to human health. Researchers have established that phytoalexins are produced in legumes under conditions of stress [7], and elicitor treatments can increase their polyphenol content. In the present study, four different legumes were induced with the food-grade fungus *A. sojae* to determine the effect on polyphenol and phytoalexin contents. The UPLC-DAD analysis of *A. sojae*-induced legumes showed the presence of different phytoalexins; however, phytoalexins were absent in the non-induced legumes (Figure 1).

The results indicated the presence of pisatin (465.95 ± 17.79 µg/g), a major phytoalexin, in the *A. sojae*-induced green pea sample. Soybean seeds treated with *A. sojae* showed high levels of glyceollins, with glyceollins I, II, and III present for a total glyceollin content of 688.86 µg/g. Phaseollin (248.59 ± 30.52 µg/g) and kievitone (386.76 ± 23.08 µg/g) were the major phytoalexins in red kidney bean. Chickpea extracts when induced with the fungus produced both maackiain (892.4 µg/g) and medicarpin (675.9 µg/g) (Table 1).

Soybean (*Glycine max* L.) has been studied well for its production of phytoalexins, and under stress, glyceollins, particularly glyceollins I, II, and III, are produced. Many different stress factors or physical stimuli induce the accumulation of glyceollins, including both abiotic and biotic inducers [43,44,45,46]. Glyceollins were initially characterized as antifungal compounds that accumulated in different soybean tissues. Research with soybeans has shown that phytoalexin accumulation increased when using *A. sojae*, *A. oryzae*, *A. niger*, and *A. flavus* [33]. The results of soybean treated with *A. sojae* indicated that the highest levels of the phytoalexins glyceollins I, II, and III occurred after incubation for three days [33]. In another study, *Aspergillus oryzae* was used as an effective inducer of glyceollins in soybean [47]. Various researchers have confirmed the production of glyceollins and other phytoalexins in soybean using both seeds and sprouts [43,48,49].

Other legumes have also been induced to produce phytoalexins that have not been studied extensively for bioactivity and health-promoting properties. Red kidney bean (*Phaseollus vulgaris* L.) when induced produced the phytoalexins kievitone and phaseollin [32,50]. Green pea (*Pisum sativum* L.) has been shown to produce pisatin as a plant defense response [51,52,53]. Another widely consumed legume that produced phytoalexins is chickpea (*Cicer arietinum* L.). Chickpeas produced the phytoalexins medicarpin and maackiain during germination [54]. Cell suspension cultures of different varieties of chickpea produced maackiain and medicarpin when induced using the blight fungus *Ascochyta rabiei* [55]. Previous research has shown that chickpeas treated with Hypnea musciformis (red algae) produced maackiain and medicarpin after incubation up to 72 h [56].

Most phytoalexins produced by legumes are isoflavones that include the pterocarpan isoflavones pisatin, medicarpin, maackiain, and glyceollins. Kievitone and phaseollin are both isoflavones produced in chickpeas. A lot of research has been conducted on identifying the biosynthetic steps and precursors produced in the production of each phytoalexin [57,58]. Pisatin was the first phytoalexin identified in green pea extracts by Cruickshank and Perrin [59]. Further research by Celoy and VanEtten elucidated the complete pathway and enzymes necessary to induce pisatin in green peas [60]. Maackiain is a precursor to the production of pisatin in peas and has been identified in some studies in pea extracts. In our study, we did not identify maackiain in samples but identified high concentrations of pisatin as the main phytoalexin produced in green peas.

### 3.2. Total Phenolics and Total Flavonoid Content

**Figure 2:**
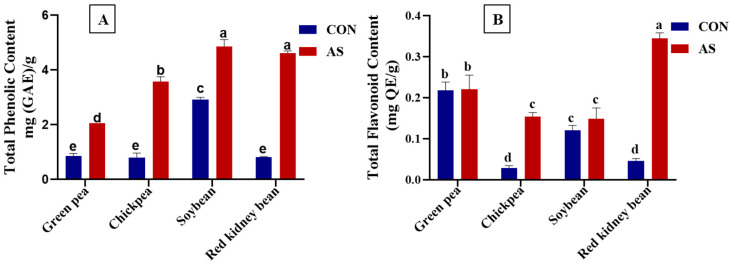
Total phenolic (**A**) and total flavonoid content (**B**) of *A. sojae*-induced and non-induced legume extracts. Results are expressed as mg (GAE)/g and mg (QE)/g for total phenolic and total flavonoid content, respectively. CON, non-induced legumes; AS, induced legumes. Data are presented as mean ± SD. For each assay, values marked with different letters indicate significant differences by Tukey’s multiple comparison test (*p* < 0.05).

The results revealed that *A. sojae*-induced extracts displayed higher total phenolic content when compared to non-induced samples. The total phenolic contents in induced and non-induced extracts of soybean, green pea, chickpea, and red kidney bean are shown in Figure 2A. The total phenolic content of induced legumes ranged from 2.05 mg (GAE)/g for GP-AS to 4.85 mg (GAE)/g for SB-AS. However, the total polyphenol content in non-induced samples ranged from 0.80 mg (GAE)/g for CP-CON to 2.92 mg (GAE)/g for SB-CON. The highest total phenolic content was reported in SB-AS. Our results are in accordance with a previous study which showed an increase in polyphenol content in *A. sojae*-treated soybean compared to the tested control [48].

The range for total flavonoid content for induced legumes was 0.14 mg (QE)/g for SB-AS to 0.34 mg (QE)/g for RKB-AS, and for non-induced legumes, it ranged from 0.02 mg (QE)/g for CP-CON to 0.21 mg (QE)/g for GP-CON, as shown in Figure 2B. Research conducted by Sharma and Giri showed that soybeans contained a higher phenolic content compared to all other tested legumes [61]. The authors revealed that soybeans, chickpeas, common beans, and peas contained 46.65, 43.65, 39.31, and 33.14 mg GAE/g total phenolics. Our investigation results are similar to those from the study conducted by Sharma and Giri [61] that showed higher phenolic content in soybean compared to all other tested legumes. Preceding research has shown that total phenolic content in the seed extracts of different cultivars of soybean ranged between 6.4 mg GAE/g and 10.5 mg GAE/g, whereas total flavonoid content was between 0.55 mg QE/g and 1.20 mg QE/g [62]. Another legume that is consumed worldwide is the kidney bean. Kan et al. [63] showed that the total phenolic and flavonoid content of different kidney bean cultivars varied between 0.25 and 3.79 mg GAE/g and 0.19 and 7.05 mg rutin equivalent/g, respectively.

### 3.3. UPLC-ESI-QTOF-MS/MS Characterization of Phenolic Acids in Legumes

The analysis of phenolic acids in legumes was achieved using UPLC-ESI-QTOF-MS/MS (Table 2). Multiple Reaction Monitoring (MRM) was utilized for the quantitation of phenolic acids with ESI (electrospray)-MS/MS. With MRM mode, the mass spectrometer monitored a specific transition according to the retention time of each phenolic acid. Each target ion generated several product ions, and quantitation was based on the most abundant product ion. The selected ion transitions, target and product ions, limits of quantitation (LOQs), and limits of detection (LODs) for each phenolic acid are reported in Table 2.

Seven phenolic acids were quantitated in *A. sojae*-induced and non-induced legume samples. Table 3 summarizes the content of individual phenolic acids present in the induced and non-induced legume samples.

**Figure 3:**
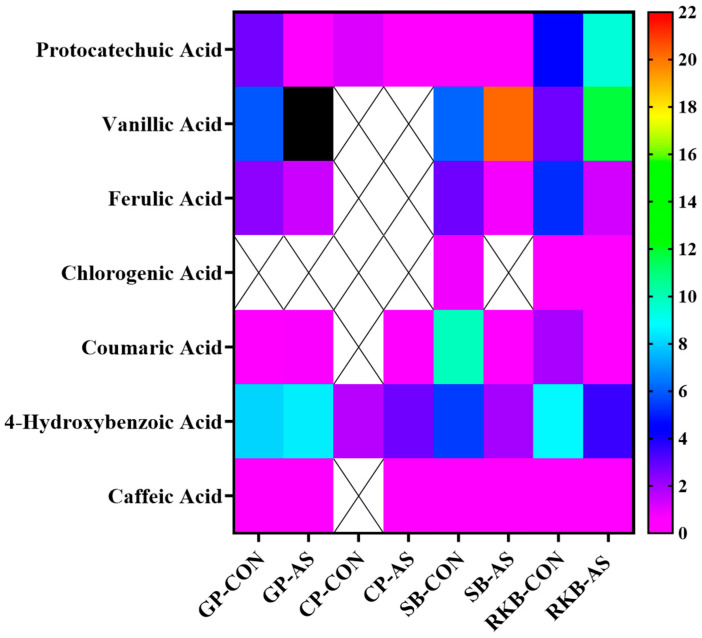
Heat map showing distribution and concentration of phenolic acids in *A. sojae*-induced and non-induced legumes. Orange-colored boxes show higher content among all samples. Black box indicates very high concentration of vanillic acid in GP-AS (out of range; range selected for heat map is 0 to 22). GP-CON, green pea non-induced; GP-AS, green pea induced; CP-CON, chickpea non-induced; CP-AS, chickpea induced; SB-CON, soybean non-induced; SB-AS, soybean induced; RKB-CON, red kidney bean non-induced; RKB-AS, red kidney bean induced.

The results revealed the presence of protocatechuic, vanillic, ferulic, chlorogenic, coumaric, 4-hydroxybenzoic, and caffeic acids in legume samples. RKB-AS and RKB-CON revealed the presence of all the targeted phenolic acids, especially vanillic acid, protocatechuic acid, and 4-hydroxybenzoic acid as the major phenolic acids. The content of phenolic acids in chickpea was relatively low, and the presence of only protocatechuic acid, coumaric acid, and 4-hydroxybenzoic acid was displayed in CP-AS. Coumaric acid was not detected in CP-CON. A heat map of the distribution and concentration of phenolic acids in *A. sojae*-induced and non-induced legumes is depicted in Figure 3. The *x*-axis of the heat map signifies samples, whereas the y-axis denotes phenolic acids. The orange color represents a higher concentration, while the pink color denotes a lower content. The content of vanillic acid was quite high in GP-AS (327.26 ± 31.86 μg/g), as represented by the black color (beyond the range) in the heat map. *A. sojae*-induced soybean showed moderate levels of vanillic acid (20.19 ± 0.97 μg/g). Other researchers have also identified phenolic acids in different legumes. Research by Wang et al. [64] showed that protocatechuic acid was a common phenolic acid in 14 common beans from China. In soybean seeds, phenolic acids were related to bitterness and astringency, and research showed antioxidant activities [65]. Chung et al. predominantly found that ferulic acid and benzoic acids were the primary phenolic acids in soybean [66].

### 3.4. Antioxidant Activity

**Figure 4:**
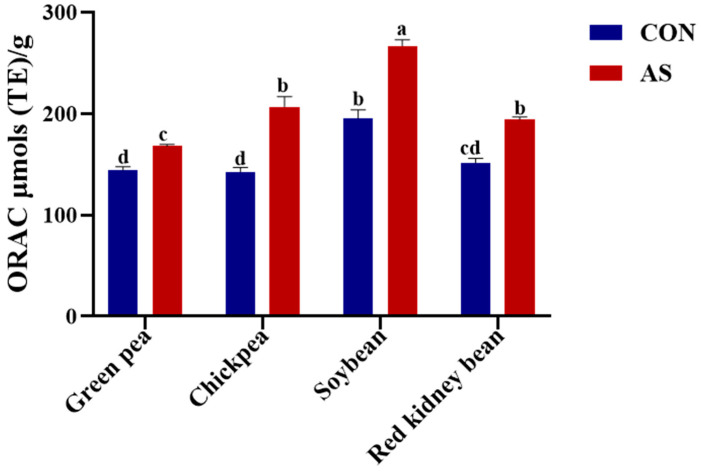
Free radical scavenging assessed by oxygen radical absorbance capacity (ORAC) assay of *A. sojae*-induced and non-induced legume extracts. Results are presented as mean ± SD. Values marked with different letters indicate significant differences by Tukey’s multiple comparison test (*p* < 0.05). CON, non-induced legumes; AS, induced legumes.

Legumes induced with *A. sojae* displayed higher levels of antioxidant activity determined using several in vitro assays. With each legume tested in our study, the levels of *A. sojae*-induced extract antioxidant activity were higher when compared to non-induced samples. The highest ORAC assay antioxidant levels shown in Figure 4 were exhibited by SB-AS (266.92 µmols TE/g) followed by CP-AS (206.86 µmols TE/g), SB-CON (195.27 µmols TE/g), and RKB-AS (194.09 µmols TE/g). The ORAC antioxidant activity of induced legumes extracts ranged from 167.86 µmols/g for GP-AS to 266.92 µmols TE/g for SB-AS. Previous research in our lab showed that soybeans treated with *A. sojae* exhibited the maximum total isoflavone levels, and the level of antioxidant activity was also higher after different treatments [67].

Other results from the DPPH and ABTS assays revealed the maximal antioxidant activity with the RKB-AS extract. Both the DPPH and ABTS assays showed a similar trend with a higher level of activity with *A. sojae*-induced samples when compared to non-induced controls. The results showed that RKB-AS exhibited 1.34 ± 0.002 mg (Trolox)/g and 5.86 ± 0.06 mg (Trolox)/g activity using the DPPH and ABTS assays, respectively. The non-induced samples of red kidney bean (RKB-CON) showed 0.92 ± 0.005 mg (Trolox)/g and 1.19 ± 0.29 mg (Trolox)/g activity using the DPPH and ABTS assays. The DPPH and ABTS results also showed a similar trend to the ORAC assay in terms of the higher level of antioxidant activity possessed by *A. sojae*-induced extracts compared to non-induced extracts. All of the induced extracts display the following order of antioxidant activity using the DPPH assay: RKB-AS > SB-AS > CP-AS > GP >AS. The results of ABTS showed the following order of antioxidant activity: RKB-AS > SB-AS > GP >AS >CP-AS. Amongst all the legumes, CP-CON and GP-CON displayed the lowest antioxidant activity in both assays. CP-CON exhibited 0.09 ± 0.0008 mg (Trolox)/g in the DPPH assay and 0.12 ± 0.07 mg (Trolox)/g in the ABTS assay, whereas GP-CON exhibited 0.15 ± 0.006 mg (Trolox)/g in the DPPH assay and 0.25 ± 0.04 mg (Trolox)/g in the ABTS assay. Table 4 presents the total antioxidant activities of the eight legume samples using the DPPH and ABTS methods.

There was a difference in the results of antioxidant activity found using the ORAC assay and both the DPPH and ABTS assays, and this difference is due to the mechanism of reaction. The ORAC method utilizes a hydrogen atom transfer and signifies a competitive reaction between an antioxidant compound and a fluorescence probe (fluorescein) for a radical [68]. The DPPH and ABTS methods are electron transfer (ET)-based methods; the antioxidant activities by both assays was assessed by the reduction of an oxidant radical by measuring the color change in the radical [68]. A lot of research has been conducted on the antioxidant activities of legumes. Legumes contain different polyphenols, including phenolic acids and flavonoids, which have antioxidant properties [14].

### 3.5. Enzyme Inhibition of A. sojae-Induced and Non-Induced Legumes

**Figure 5:**
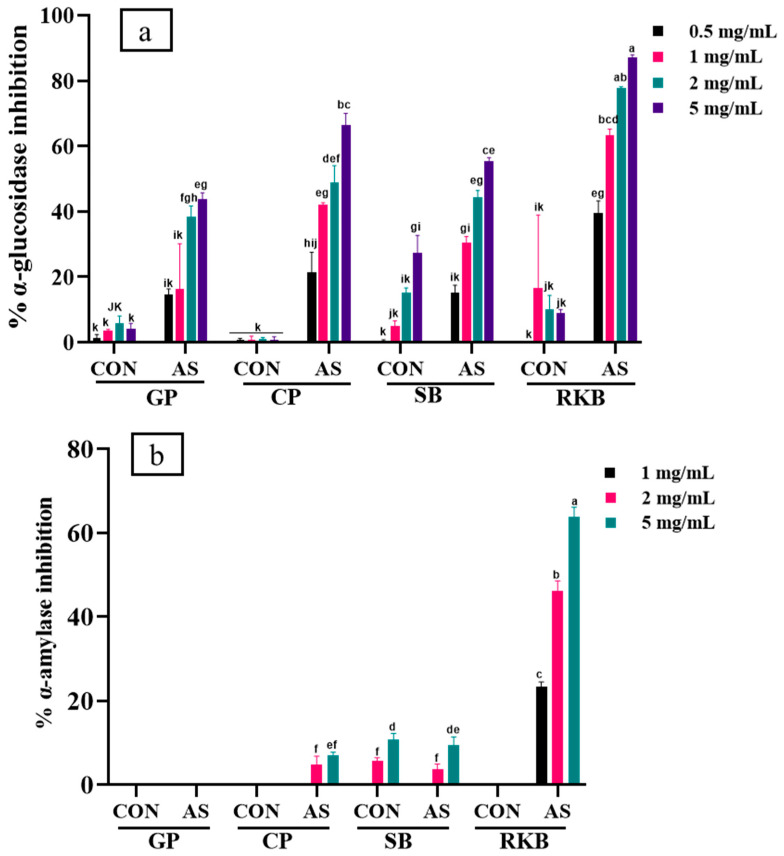
α-glucosidase (**a**) and α-amylase (**b**) inhibitory effects of *A. sojae*-induced and non-induced legume extracts at different concentrations. Results are presented as mean ± SD. Values marked with different letters indicate significant differences by Tukey’s multiple comparison test (*p* < 0.05). GP-CON, green pea non-induced; GP-AS, green pea induced; CP-CON, chickpea non-induced; CP-AS, chickpea induced; SB-CON, soybean non-induced; SB-AS, soybean induced; RKB-CON, red kidney bean non-induced; RKB-AS, red kidney bean induced.

In this study, the α-amylase and α-glucosidase inhibitory potentials of *A. sojae*-treated and untreated soybean, chickpea, green pea, and red kidney beans were evaluated using enzyme inhibitory assays. *A. sojae*-induced legume extracts inhibited the α-glucosidase enzyme and displayed inhibition activities from 14.5 to 87.16% from 0.5 to 5 mg/mL. However, non-induced legumes displayed the lowest level of inhibitory activity at all the concentrations tested. Among the legumes, red kidney bean (RKB-AS) revealed the highest level of α-glucosidase activity at all the selected concentrations with the highest level of activity observed at 5 mg/mL (87.16%). The lowest level of α-glucosidase activity was displayed by green pea (GP-CON) and chickpea (CP-CON) at all the tested concentrations. The results of α-glucosidase inhibition are shown in Figure 5a. Similarly, the levels of α-amylase’s inhibitory effects were also higher in *A. sojae*-induced legume samples. Red kidney bean (RKB-AS) exhibited the highest level of α-amylase activity of all the legumes tested. In contrast with the induced samples (RKB-AS), non-induced (RKB-CON) samples did not show inhibition activity at over the sample concentrations used, as shown in Figure 5b. The main difference between RKB-AS and RKB-CON is the production of the phytoalexins kievitone (386.76 μg/g) and phaseollin (348.59 μg/g) in RKB-AS. These two isoflavones may contribute to amylase and glucosidase inhibition in the RKB-AS extract. Additionally, protocatechuic acid and vanillic acid were also at higher levels in RKB-AS when compared to RKB-CON. Soybean SB-CON showed α-glucosidase activity; however, the SB-AS extract displayed a higher level of α-glucosidase activity particularly at the higher doses used in this study. Glyceollin I was determined to be present at high concentrations in SB-AS at 441.43 μg/g. Previous research has determined that the glyceollins inhibit α-glucosidase [69]. Chickpea CP-CON showed the inhibition of α-glucosidase; however, a high level of inhibitory activity was determined using the CP-AS extract. Research by Dendup et al. showed that medicarpin inhibited α-glucosidase [70]. Maackiain may also contribute to the enzyme inhibition activity shown. Green pea (induced and non-induced) did not display any α-amylase activity at the concentrations used in this study. A lot of research has examined legumes as a source of compounds to inhibit the digestive enzymes α-glucosidase and α-amylase. Research has shown that polyphenolic antioxidants in mung and adzuki beans inhibited α-glucosidase [16]. Polyphenol extracts from adzuki, lima, mung, and pinto beans displayed inhibitory effects against α-glucosidase and lipase [71]. In this study, non-induced red kidney bean extracts had the maximal level of α-glucosidase activity at 1 mg/mL. However, *A*. *sojae*-induced red kidney bean extracts showed a significantly higher level of inhibitory activity at each dose tested when compared to the non-induced extracts. In research by Tan et al., black soybean extracts and fractions showed abilities to inhibit α-amylase, and black turtle bean extracts were able to inhibit α-glucosidase [31].

**Figure 6:**
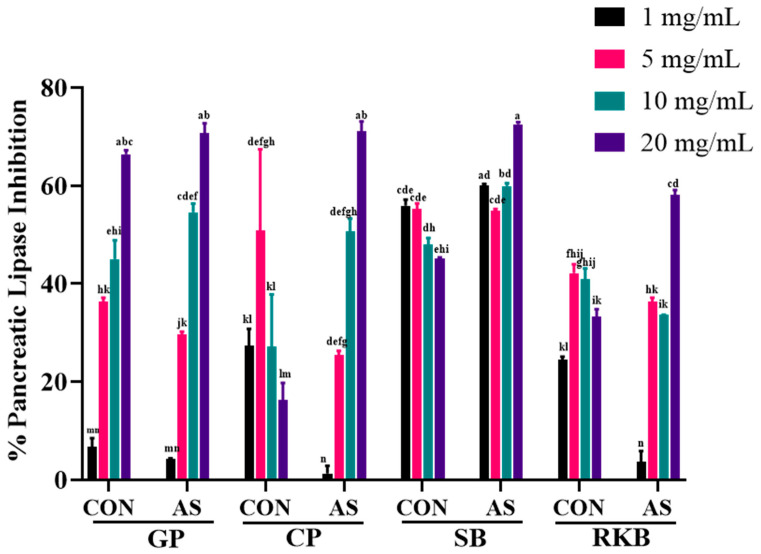
Pancreatic lipase inhibitory effects of *A. sojae*-induced and non-induced legume extracts at 1, 5, 10, and 20 mg/mL concentrations. Results are presented as mean ± SD. Values marked with different letters indicate significant differences by Tukey’s multiple comparison test (*p* < 0.05). GP-CON, green pea non-induced; GP-AS, green pea induced; CP-CON, chickpea non-induced; CP-AS, chickpea induced; SB-CON, soybean non-induced; SB-AS, soybean induced; RKB-CON, red kidney bean non-induced; RKB-AS, red kidney bean induced.

Pancreatic lipase has been shown to be a vital digestive enzyme that aids in the digestion of dietary lipids and converts triglycerides to free fatty acids and monoglycerides. Blocking pancreatic lipase is directly associated with reduced cholesterol concentrations in the body. Therefore, lipase inhibitors can be used as source for the development of sustainable antiobesity dietary supplements. Almost all the legumes inhibited pancreatic lipase at all the tested concentrations (1, 5, 10, and 20 mg/mL) (Figure 6). Biosynth (Berkshire, UK).

The highest levels of inhibitory effects were observed at higher concentrations (20 mg/mL); however, legumes also showed prominent inhibitory effects at 5 and 10 mg/mL. The maximum levels of lipase inhibitory effects were displayed by SB-AS (72.54%), CP-AS (71.09%), and GP-AS (70.68%) at 20 mg/mL. All the *A. sojae*-induced legumes exhibited higher levels of inhibitory effects compared to non-induced legumes. Past research on inhibiting pancreatic lipase has shown that legume extracts without induction have inhibitory activities [72]. Both black soybean and turtle bean extracts displayed comparable inhibitory effects against lipase [31]. In research by Lee et al., there were no significant dietary differences between the median inhibitory activity of seven legumes, including black soybean and chickpeas [72]. In research by Hong et al., a soybean leaf extract inhibited lipase using a fluorometric assay, and several flavonoids (genistein, daidzein, formononetin, axifolin, diosmetin, and glycitein) were identified by UPLC-MS/MS analysis as active components [73]. In our testing, constitutive soybean isoflavones contribute to lipase inhibition in SB-CON, and these isoflavones combined with the induced glyceollins contribute to the lipase inhibitory activities of the *A. sojae*-induced soybeans. This same process occurs in each legume sample tested, where control seeds have polyphenols, and these polyphenols are combined with additional phytoalexins after induction with *A. sojae*. Today, legume polyphenols and processing methods for increasing the levels of bioactivity have emerged as important topics in food science. Our study indicates that legumes induced by *A. sojae* are a source of health-promoting beneficial components.

## 4. Conclusions

In conclusion, *A. sojae*-induced legumes showed increased phytoalexin and polyphenol content and higher levels of antioxidant activity when compared with control legumes. Amongst all the legumes, induced red kidney bean and soybean displayed higher levels of antioxidant activity. The antidiabetic effects of soybean, green pea, chickpea, and red kidney bean with and without *A. sojae* induction were characterized, and red kidney beans induced with *A. sojae* had the highest α-glucosidase and α-amylase inhibition activity levels. In addition, the results obtained from enzyme inhibitory assays provide insights into the beneficial effects of different legumes with induction with the biotic elicitor *A. sojae*. The present study highlights that *A. sojae* efficiently produced phytoalexins in various legumes and could be utilized as a sustainable source for producing phytoalexin-enriched foods. According to our hypothesis, *A. sojae*-induced legumes have potential as innovative sources of pancreatic lipase inhibitors and antidiabetic and antiobesity agents.

## Figures and Tables

**Table 1:** The content of different phytoalexins produced in *A. sojae*-induced legumes.

Extracts	Phytoalexins	Content (µg/g DW)	Molecular Formula	Molecular Weight (g/mol)	Absorbance
CP-AS	Maackiain	892.40 ± 23.98 ^a^	C_16_H_12_O_5_	284.26	310
	Medicarpin	675.94 ± 46.64 ^b^	C_16_H_14_O_4_	270.27	285
GP-AS	Pisatin	465.95 ± 17.79 ^c^	C_17_H_14_O_6_	314.293	310
SB-AS	Glyceollin III	93.615 ± 4.636 ^g^	C_20_H_18_O_5_	338.4	285
	Glyceollin II	153.82 ± 8.895 ^f^	C_20_H_18_O_5_	338.4	285
	Glyceollin I	441.43 ± 7.589 ^cd^	C_20_H_18_O_5_	338.4	285
RKB-AS	Kievitone	386.76 ± 23.08 ^d^	C_20_H_20_O_6_	356.4	292
	Phaseollin	248.59 ± 30.52 ^e^	C_20_H_18_O_4_	322.36	285

Results are expressed as µg/g DW. Results are presented as mean ± SD. Values marked with different letters (^a–g^) indicate significant differences by Tukey’s multiple com-parison test (*p* < 0.05). GP-AS, green pea induced; CP-AS, chickpea induced; SB-AS, soybean induced; RKB-AS, red kidney bean induced.

**Table 2:** Identification of targeted phenolic acids by UPLC-ESI-QTOF-MS/MS.

Phenolic Acids	Molecular Formula	RT (min)	Mode of Ionization	Molecular Weight	Target (Precursor) Ion [M − H]^−^ (*m*/*z*)	Product Ion 1 (*m*/*z*)	Product Ion 2 (*m*/*z*)	LOQ (µg/mL)	LOD (µg/mL)	Samples
Protocatechuic Acid	C_7_H_6_O_4_	3.4	[M − H]^−^	154.02	153.0293	109.0354		0.005	0.002	GP-CON, GP-AS, CP-CON, CP-AS, SB-CON, SB-AS, RKB-CON, RKB-AS
Vanillic Acid	C_8_H_8_O_4_	4.40	[M − H]^−^	168.14	167.0442	108.0284	123.0439	0.100	0.050	GP-CON, GP-AS, SB-CON, SB-AS, RKB-CON, RKB-AS
Ferulic Acid	C_10_H_10_O_4_	5.07	[M − H]^−^	194.05	193.0601	134.0442	178.0371	0.100	0.050	GP-CON, GP-AS, SB-CON, SB-AS, RKB-CON, RKB-AS
Chlorogenic Acid	C_16_H_18_O_9_	4.10	[M − H]^−^	354.09	353.1069	191.0680	174.9671	0.005	0.002	SB-CON, RKB-CON, RKB-AS
Coumaric Acid	C_9_H_8_O_3_	5.01	[M − H]^−^	164.04	163.0491	119.0572		0.003	0.002	GP-CON, GP-AS, CP-AS, SB-CON, SB-AS, RKB-CON, RKB-AS
4-Hydroxybenzoic Acid	C_7_H_6_O_3_	4.01	[M − H]^−^	138.03	137.0310	93.0400		0.010	0.005	GP-CON, GP-AS, CP-CON, CP-AS, SB-CON, SB-AS, RKB-CON, RKB-AS
Caffeic Acid	C_9_H_8_O_4_	4.30	[M − H]^−^	180.04	179.0310	135.0520		0.002	0.001	GP-CON, GP-AS, SB-CON, SB-AS, RKB-CON, RKB-AS

**Table 3:** Phenolic acid profiling of *A. sojae*-induced and non-induced legume extracts.

Phenolic Acids (µg/g)	Green Pea		Chickpea		Soybean		Red Kidney Bean	
	CON	AS	CON	AS	CON	AS	CON	AS
Protocatechuic Acid	2.67 ± 0.26	0.37 ± 0.03	1.16 ± 0.04	0.10 ± 0.04	0.53 ± 0.20	0.43 ± 0.05	4.49 ± 0.28	9.44 ± 0.76
Vanillic Acid	5.87 ± 0.69	327.26 ± 31.86	-	-	6.13 ± 0.82	20.19 ± 0.97	2.76 ± 0.62	11.78 ± 0.69
Ferulic Acid	2.30 ± 0.43	1.40 ± 0.09	-	-	2.75 ± 0.30	0.78 ± 0.08	5.20 ± 1.12	01.31 ± 0.22
Chlorogenic Acid	-	-	-	-	0.81 ± 0.05	-	0.62 ± 0.02	0.56 ± 0.01
Coumaric Acid	0.58 ± 0.11	0.67 ± 0.07	-	0.10 ± 0.02	9.89 ± 1.23	0.42 ± 0.02	1.91 ± 0.24	0.40 ± 0.03
4-Hydroxybenzoic Acid	8.05 ± 2.39	8.46 ± 0.16	1.69 ± 0.09	2.73 ± 0.57	5.46 ± 0.58	1.95 ± 0.12	8.71 ± 1.08	3.52 ± 0.12
Caffeic Acid	0.04 ± 0.01	0.05 ± 0.01	-	-	0.02 ± 0.01	0.03 ± 0.01	0.10 ± 0.03	0.10 ± 0.01
Total	19.51	338.21	2.85	2.93	25.59	23.8	23.79	27.11

Results are expressed as µg/g DW. Results are presented as mean ± SD. CON = non-induced legumes; AS = induced legumes.

**Table 4:** Free radical scavenging effects of *A. sojae*-induced and non-induced legume extracts using DPPH and ABTS assays.

Legumes		ABTS mg (Trolox)/g	DPPH mg (Trolox)/g
Green pea	CON	0.25 ± 0.040 ^f^	0.15 ± 0.006 ^g^
	AS	3.01 ± 0.021 ^c^	0.39 ± 0.010 ^f^
Chickpea	CON	0.12 ± 0.07 ^f^	0.09 ± 0.0008 ^h^
	AS	2.78 ± 0.08 ^c^	0.51 ± 0.006 ^e^
Soybean	CON	1.87 ± 0.20 ^d^	0.65 ± 0.02 ^d^
	AS	3.76 ± 0.12 ^b^	0.82 ± 0.01 ^c^
Red kidney bean	CON	1.19 ± 0.29 ^e^	0.92 ± 0.005 ^b^
	AS	5.86 ± 0.06 ^a^	1.34 ± 0028 ^a^

Results are presented on a dry weight (DW) basis and expressed as mg (Trolox)/g. For each assay, values marked with different letters (^a–h^) indicate significant differences by Tukey’s multiple comparison test (*p* < 0.05). Values are mean ± SD (n = 3). CON = non-induced legumes; AS = induced legumes.

## Data Availability

The original contributions presented in the study are included in the article, further inquiries can be directed to the corresponding author.
